# Second generation multiple reaction monitoring assays for enhanced detection of ultra-low abundance *Mycobacterium tuberculosis* peptides in human serum

**DOI:** 10.1186/s12014-017-9156-y

**Published:** 2017-06-05

**Authors:** Carolina Mehaffy, Karen M. Dobos, Payam Nahid, Nicole A. Kruh-Garcia

**Affiliations:** 1 Department of Microbiology, Immunology and Pathology, Colorado State University, 1682 Campus Delivery, Fort Collins, CO 80524 USA; 2 Proteomics and Metabolomics Facility, Colorado State University, 2021 Campus Delivery, Fort Collins, CO 80523 USA; 3 Division of Pulmonary and Critical Care Medicine, University of California, San Francisco, San Francisco, CA 94143 USA

**Keywords:** MRM (Multiple Reaction Monitoring), Mass Spectrometry, Tuberculosis, Exosomes, *Mycobacterium tuberculosis*, Biomarker

## Abstract

**Background:**

*Mycobacterium tuberculosis (Mtb)* is the causative agent of Tuberculosis (TB), the number one cause of death due to an infectious disease. TB diagnosis is performed by microscopy, culture or PCR amplification of bacterial DNA, all of which require patient sputum or the biopsy of infected tissue. Detection of mycobacterial products in serum, as biomarkers of diagnosis or disease status would provide an improvement over current methods. Due to the low-abundance of mycobacterial products in serum, we have explored exosome enrichment to improve sensitivity. *Mtb* resides intracellularly where its secreted proteins have been shown to be packaged into host exosomes and released into the bloodstream. Exosomes can be readily purified assuring an enrichment of mycobacterial analytes from the complex mix of host serum proteins.

**Methods:**

Multiple reaction monitoring assays were optimized for the enhanced detection of 41 *Mtb* peptides in exosomes purified from the serum of individuals with TB. Exosomes isolated from the serum of healthy individuals was used to create and validate a unique data analysis algorithm and identify filters to reduce the rate of false positives, attributed to host *m*/*z* interference. The final optimized method was tested in 40 exosome samples from TB positive patients.

**Results:**

Our enhanced methods provide limit of detection and quantification averaging in the low femtomolar range for detection of mycobacterial products in serum. At least one mycobacterial peptide was identified in 92.5% of the TB positive patients. Four peptides from the *Mtb* proteins, Cfp2, Mpt32, Mpt64 and BfrB, show normalized total peak areas significantly higher in individuals with active TB as compared to healthy controls; three of the peptides from these proteins have not previously been associated with serum exosomes from individuals with active TB disease. Some of the detected peptides were significantly associated with specific geographical locations, highlighting potential markers that can be linked to the *Mtb* strains circulating within each given region.

**Conclusions:**

An enhanced MRM method to detect ultra-low abundance *Mtb* peptides in human serum exosomes is demonstrated, highlighting the potential of this methodology for TB diagnostic biomarker development.

**Electronic supplementary material:**

The online version of this article (doi:10.1186/s12014-017-9156-y) contains supplementary material, which is available to authorized users.

## Background

The World Health Organization estimates that 2 billion people globally are infected with *Mycobacterium tuberculosis (Mtb)* [1]. One of the biggest hurdles for the global control of tuberculosis (TB) is the lack of point-of-care, rapid and accurate diagnostic tools. Current diagnostics rely on sputum availability to confirm the presence of bacteria by microscopy, live bacilli by culture or using molecular tools to detect pathogen DNA. However, microscopy only detects 20–80% of all active TB cases [2] and although diagnosis by sputum culture is highly sensitive, it can take 4–6 weeks to yield results; this results in delays in treatment and continued transmission of disease in the community. To reduce rates of TB worldwide, simpler more easily scalable accurate diagnostics are needed, prompting research on novel biomarker discovery and new diagnostic assays, with an emphasis on alternative non-sputum diagnostics targeting blood, urine, and breath.

We previously demonstrated that during cellular infection with *Mtb*, mycobacterial products can be incorporated into host cell exosomes [3]; the presence of *Mtb* proteins were confirmed in exosomes isolated from a variety of fluids from infected animal models [4, 5]. Exosomes are 100 nm vesicles generated by all nucleated cell types and released into biofluids, including blood, urine and sputum [6–10]. Purification of exosomes from serum is a facile means to reduce the complexity of the fluid and concentrate the encapsulated *Mtb* proteins. Despite the simplification, the exosomes of interest, that is those derived from the *Mtb*-infected cells, are still a minor component of the entire exosome population. Therefore coupling this purification with a sensitive downstream detection platform is critical for the detection of mycobacterial proteins as potential biomarker candidates.

Using targeted mass spectrometry we previously conducted a pilot study to determine if 33 mycobacterial proteins that we previously identified in cell culture [3] and animal studies were also present in exosomes purified from the serum of individuals diagnosed with active disease or known to have latent TB infection [11]. We found at least one of the 76 peptides from 27 of the 33 *Mtb* proteins in at least a single individual of a cohort or 57 subjects; the remaining 6 *Mtb* proteins were not identified in any of the 57 samples. This discovery experiment provided us with a preliminary list of *Mtb* protein candidate biomarkers and a rapid method for triaging peptide candidates to proceed to assay refinement and larger verification studies.

The main aim of this study was to enhance an MRM method to detect *Mtb* peptides in serum exosomes from TB patients. The aforementioned study by our group used 17 unrefined targeted MRM assays to screen for the presence of peptides from 33 *Mtb* proteins [11]. The goal of this study is to validate these findings using refined MRM methods to reduce the number of channels being monitored and improve sensitivity and specificity by spiking isotopically labeled peptide standards and including control samples from healthy donors. Novel data analysis methodology was applied to confirm the presence of 19 low-abundance mycobacterial proteins in samples dominated by host proteins. In addition, in this study we have analyzed samples from both HIV+ and HIV− TB patients. Individuals with HIV/TB co-infection are often difficult to diagnose using current techniques due to paucibacillary presentation of the disease which can compromise detection by microscopy, and while detection by culture has higher positivity rates, the collection of several samples is often needed to achieve a conclusive diagnosis in these patients [12, 13]. HIV+ infected patients also have a lower rate of positivity using the TB skin test or Interferon Gamma Release Assays due to their inherent immunocompromised status [13–15]. Thus, a diagnostic method that can efficiently detect both HIV+ and HIV− TB disease and/or TB infection at similar rates is needed. The ultimate goal is to define biomarkers of active disease (regardless of HIV status), discovered and verified by MRM-MS, and to translate the promising candidates to a point-of-care platform for use in the field that is independent of complex sample processing and high-end instrumentation.

## Methods

### Study design

Serum samples from TB negative (suspect) and TB positive patients were obtained from the Foundation for Innovative Novel Diagnostics (FIND) specimen repository (Geneva, Switzerland). Serum samples from healthy donors, with no history of tuberculosis were purchased from Bioreclamation IVT (Westbury, NY). All samples were stored at −80 °C upon arrival until processed. For initial method development and optimization we used 16 TB negative and 20 TB positive serum samples from 4 different geographic locations (Table 1); these samples were pooled and used as background matrix. For MRM method validation we used 20 individual healthy controls and 40 individual TB positive serum samples all with culture confirmed pulmonary tuberculosis (Table 2).

**Table 1:** Patient breakdown of samples included in the pooled matrix sample

TB status	Smear status	HIV status	Geography
TB positive, *n* = 20	Positive, *n* = 12 negative, *n* = 8	Positive, *n* = 8 negative, *n* = 12	Bangladesh, *n* = 5 South Africa, *n* = 5 Peru, *n* = 5 Vietnam, *n* = 5
TB negative, *n* = 16		Positive, *n* = 4 negative, *n* = 12	Bangladesh, *n* = 4 South Africa, *n* = 5 Peru, *n* = 4 Vietnam, *n* = 1

**Table 2:** Patient breakdown of samples included in the assay verification set

TB status	Smear status	HIV status	Geography
TB positive, *n* = 40	Positive, *n* = 22 negative, *n* = 18	Positive, *n* = 13 negative, *n* = 27	Bangladesh, *n* = 10 South Africa, *n* = 10 Peru, *n* = 10 Vietnam, *n* = 10

### Sample processing

Serum samples (250 µL) were centrifuged to remove whole cells/cellular debris. Exoquick (System Biosciences, Palo Alto, CA) was added to the cleaned serum at a 4:1 ratio (sample:reagent), incubated at 4 °C for 30 min, and exosomes were pelleted by centrifugation at 1.5 k x g for 30 min, as per manufacturer recommendation. The exosomes were suspended in 250 µL of PBS and micro bicinchoninic acid assay was performed to quantify protein content. To construct the pooled matrix stock, 50 µg (protein) of purified exosomes from the 36 samples listed in Table 1, were mixed. 20 µg of pooled matrix or 50 µg (1.18 ± 0.41 µL) of individual exosome sample were run into a NuPAGE Novex 4-12% Bis–Tris Gel 1.0 mm gel for 5 min in NuPAGE MES SDS Running Buffer (Life Technologies, Carlsbad, CA) to trap the Exoquick polymer prior to peptide extraction. In-gel digest with sequencing-grade trypsin (Roche, Switzerland) was performed at a 1:20 (enzyme:substrate) ratio, overnight at 37 °C, as previously described [3]. The extracted peptides were dried and suspended at a final concentration of 1 µg/µL in 3% acetonitrile (ACN), 0.5% formic acid (FA) in water.

### Peptide standards

Isotope-labelled standards for each of our peptides of interest were purchased from New England Peptide (Gardner, MA). All peptides were prepared to a minimum of 95% purity and confirmed by HPLC. QC of chromatography was monitored by the addition of indexed retention time (iRT) standards (Biognosys AG, Switzerland) [16]. 10 nM mixes of the isotope-labelled peptide standards and iRT mix at a 0.4× final concentration were spiked into each sample.

### Multiple reaction monitoring

Daily Skyline (64-bit) was used to build and optimize the multiple reaction monitoring (MRM) methods for the relative quantification of peptides [17]. For this study, two MRM methods were built in Skyline. The first (MRM-1), included 20 peptides from 9 *Mtb* proteins (Table 3). The second method (MRM-2), included 10 *Mtb* proteins and 21 peptides (Table 3). Briefly, FASTA-formatted sequences of all 19 proteins were used for in silico tryptic (KR|P) digestion with peptides being selected based on previous discovery studies [4, 18]. Both double and triple charge precursor ions were empirically tested and selected based on their performance. “y” ions for each transition were selected based on a library built from LC–MS/MS data acquired in an Orbitrap Velos (Thermo Scientific) (Additional file 1). The resultant methods were exported to Masslynx (Waters Corporation, Milford, MA). All method development was performed using a 10 nM mix of all 41 heavy labeled peptides (K^ = Lysine, 13C6, 15N2 or R^ = Arginine, 13C6, 15N4) (New England Peptide, Gardner, MA) in a 1 µg/µL matrix background (as described above). One and a half microliters (1.5 µL) of the heavy labeled peptide mix in background matrix were then injected into the LC–MS/MS system consisting of a Waters nanoACQUITY UPLC coupled to a Waters Xevo TQ-S mass spectrometer fitted with a Trizaic source. The instrument was operated with MassLynx V4.1 SCN810 (Waters Corporation, Milford, MA). Chromatography was performed on a 150 µm × 50 mm Ion key packed with BEH C18 130 Å, 1.7 um. The chromatography length and gradient were optimized to separate all peptides as much as possible so that at least 12 points per each transition were acquired. Peptides were separated using gradient elution with a stable flow rate of 3.06 µL/min. A linear method consisting of 2 min of equilibration in 97% buffer A (99.9% water with 0.1% formic acid) and 3% buffer B (99.9% ACN with 0.1% formic acid), followed by a 45 min linear gradient to 22% buffer B. The method finished with 5 min wash at 97% buffer B and final equilibration at 3% buffer B for 5 min. The column was maintained at 45 °C during analysis, and the samples were kept at 4 °C at all times. The mass spectrometer was operated in selective reaction monitoring mode using electrospray ionization in nanospray positive ion mode, with a capillary voltage of 3.6 kV and a source temperature of 100 °C. Cone voltage was static at 35 V and the collision energies were in silico predicted by Skyline for each compound individually (Additional file 1). The final methods included the selection of the 5 most abundant transitions per peptide and the precursor ion with the best peak shape and overall signal.

**Table 3:** Peptides/proteins included in MRM assays 1 and 2

Name	Rv#	Peptide	Assay	Name	Rv#	Peptide	Assay
AcpM	Rv2244	IPDEDLAGLR	2	GlcB	Rv1837c	VVADLTPQNQALLNAR	1
		TVGDVVAYIQK	2			FALNAANAR	1
		LEEENPEAAQALR	2			NYTAPGGGQFTLPGR	1
Ag85a	Rv3804c	NDPLLNVGK	2	GlnA1	Rv2220	SVFDDGLAFDGSSIR	2
		FLEGFVR	2			GGYFPVAPNDQYVDLR	2
Ag85b	Rv1886c	PGLPVEYLQVPSPSMGR	2	GroES	Rv3418c	DVLAVVSK	1
		AADMWGPSSDPAWER	2			RIPLDVAEGDTVIYSK	1
Ag85c	Rv0129c	VQFQGGGPHAVYLLDGLR	1	HspX	Rv2031c	AELPGVDPDK	1
		NDPMVQIPR	1			TVSLPVGADEDDIK	1
		FLEGLTLR	1	Mpt32	Rv1860	TTGDPPFPGQPPPVANDTR	1
BfrB	Rv3841	EALALALDQER	1			LYASAEATDSK	1
		AGANLFELENFVAR	1	Mpt64	Rv1980c	SLENYIAQTR	1
Cfp2	Rv2376c	GSLVEGGIGGTEAR	1			FLSAATSSTPR	1
		SLADPNVSFANK	1	PpiA	Rv0009	IALFGNHAPK	2
Cfp10	Rv3874	QELDEISTNIR	2			VIQGFMIQGGDPTGTGR	2
DnaK	Rv0350	TTPSIVAFAR	2			HTIFGEVIDAESQR	2
		ITQDLLDR	2	MrsA	Rv3441c	YVLEELR	2
Esat-6	Rv3875	LAAAWGGSGSEAYQGVQQ	2			TAVEQAAAELGDTGR	2
		WDATATELNNALQNLAR	2	SahH	Rv3248c	GVTEETTTGVLR	1
GarA	Rv1827	FLLDQAITSAGR	2			IHVEALGGHLTK	1
		LVFLTGPK	2				

### MRM of clinical samples

Trypsin digested clinical samples were resuspended at a final concentration of 1 µg/µL in 5% Acetonitrile, 0.1% Formic acid containing 10 nM internal standard mix. After resuspension each sample was centrifuged 10 min to pellet minor impurities. Supernatant was then transferred to a MS vial and placed in the Xevo-TQS autosampler. One and a half microliters (1.5 µL) were injected into the instrument and data was acquired as described above monitoring for both heavy and light forms for a total of 10 transitions per peptide. Blank runs were run every 4 samples. After MRM analysis of each clinical sample, raw files were imported into Skyline where prior to data processing in Excel (see section below), each sample was manually validated for quality (i.e. retention time; peak shape; and intensity and peak boundaries of internal standards). The majority of samples were run only once. However in cases were the sample did not pass the manual QC (usually due to retention time drift), that sample was re-injected at least once. In some cases, even after multiple injections, data did not pass manual QC. In those case that particular sample/peptide was removed from the results.

### Determination of limit of detection (LOD) and limit of quantification (LOQ)

Serial dilutions (10–0.01 nM) of the heavy labeled peptides were prepared in 1 µg/µL of pooled matrix. 1.5 µL of each dilution were injected into the LC–MS/MS system described above using a 2 or 4 min window for acquisition of precursor and transition of each peptide. All dilution points were run in triplicate. Raw data resulting from each of the dilution points were exported into Skyline. Peaks were manually validated and boundaries adjusted if necessary. Only the final set of transitions after validation with clinical samples was used for LOD/LOQ purposes. Area under the peak for each of the validated transitions was exported into excel and the LOD/LOQ was calculated using the LINEST method. Briefly, a linear regression was performed and the LOD and LOQ were calculated as shown in Eqs. 1 and 2 respectively, where m is the slope of the curve and *s*(*y*) is the standard deviation of the *y* values.

1 $${\text{LOD}} = 3*\frac{m}{s\left( y \right)}$$

2 $${\text{LOQ}} = 10*\frac{m}{s\left( y \right)}$$

All LOD and LOQ values are expressed in fmol/µL (Additional file 2).

### Liquid chromatography-multiple reaction monitoring mass spectrometry analysis

Data processing was performed using Skyline software. Manual inspection and border adjustment of isotopic standard peaks was performed for each peptide. While five transitions were monitored for each peptide, the final analysis used 3–5 transitions selected based on low background/noise level. Any standard peptide which showed either an aberrant transition ratio or retention time (RT) was used to disqualify the inclusion of the native peptide from sample analysis.

### Data post-processing

**Fig. 1:**
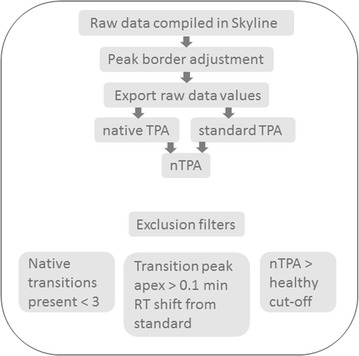
Raw data processing workflow. *TPA* total peak area (sum of transition peaks), *nTPA* normalized TPA (ratio native/labeled standard), *TPA* healthy cut-off determined by mean +3× SD

After initial screening in Skyline, peak areas for individual transitions and RTs for both native and standard peaks were exported to Excel. In total 19 methods for processing the raw data were trialed (data not shown). The final method, summarized in Fig. 1, utilized the sum of the transition peak areas or total peak area (TPA) for each native peptide, followed by normalization by the TPA of the isotope-labelled standard (peak area ratio). In order for a peak to be qualified for inclusion, three minimal qualifications were set: (1) the TPA of the native peptide must be composed of values from 3 or more transitions, (2) the RT of native transition peaks must be within 0.1 min of the standard transition peak, and (3) the final normalized TPA must exceed the cut-off determined by the healthy donor samples. The post data-processing protocol, including the first two qualifications for inclusion stated above, were applied to the raw values for the healthy data to formulate the normalized TPAs. The healthy cut-off value was established as the mean normalized TPA plus three times the standard deviation of all 20 samples and are summarized in Additional file 3. False discovery rates, calculated as the percent of healthy samples with nTPA values above the cut-off is included in Additional file 3; the maximal number of controls with a positive signal in any sample is 1 (5% FDR). The final Skyline data files have been deposited to the Panorama Repository (https://panoramaweb.org/) [19]. Unpaired, two-tailed t-tests were performed using GraphPad Prism version 6.00 for Windows (La Jolla, CA). Benjamin-Hochberg adjustment was used to control the false discovery rate.

## Results

### LOD/LOQ determination in a pooled exosome matrix

Exosomes isolated from the serum of 16 TB suspects and 20 TB culture-confirmed patients were pooled to create a matrix for the determination of the LOD and LOQ for the 41 peptides in our two MRM assays. Most peptides displayed sub-nanomolar (nM) LODs (Additional file 2). The rationale for creating a pooled matrix for the purposes of establishing LOD and LOQ was to maximize the likelihood of selecting the most intense transitions for query as we have previously identified several transitions which are highly influenced by the matrix (data not shown), and also to reduce bias created by a single representative sample. Individual transitions were subject to elimination from final analyses if LOD was greater than 1 nM, with a maximum removal of two transitions per peptide.

### Identification and geographic diversity of *Mtb* peptides in serum exosomes

**Fig. 2:**
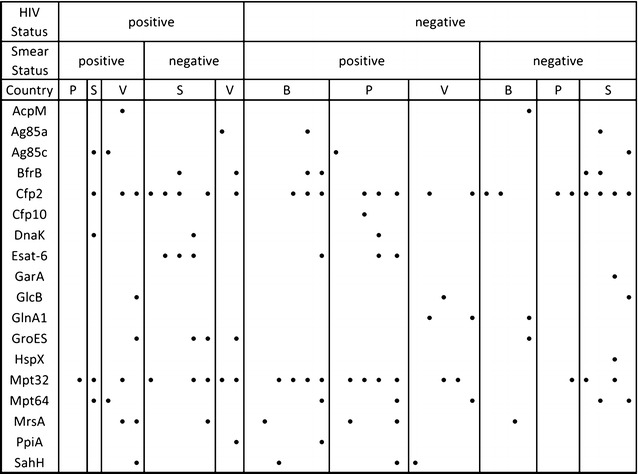
Stratification of patient samples by HIV status, sputum smear microscopy status, and geography. *Each column* indicates a single patient (*n* = 40). *Black circles* indicate the presence of each protein (*rows*) determined by a normalized TPA of one or more peptides above the healthy threshold. Geography is indicated by: *B* Bangladesh, *P* Peru, *S* South Africa, *V* Vietnam

Out of 40 patients with active TB, we were able to identify 35 (87.5%) of them by the identification of at least one *Mtb* peptide monitored in MRM-1 in their serum exosomes. MRM-2 was less successful, allowing for the identification of only 23 (57.5%) of the TB positive patients. Overall, if both assay 1 and 2 results are combined, we see at least one peptide present in 37 out of the 40 active TB subjects (92.5%). The gain of 2 new positive IDs were based on peptide TAVEQAAAELGDTGR from the *Mtb* protein MrsA (Rv3441c) from MRM-2. Three patients out of the 40 (7.5%) did not have any bacterial peptide identified by either MRM assay, and they varied by smear status, HIV status, and region of origin (Fig. 2). Sixteen out of 17 (94%) patients missed by sputum smear microscopy were identified in our MRM assays by at least one peptide (Fig. 2; Additional file 4). There was no statistical difference (p = 0.14) between the numbers of peptides identified in HIV positive patients (3.2 ± 1.7) when compared to those without co-infection (2.4 ± 1.6). When stratified by geographical location, patients from South Africa displayed the highest numbers of peptides identified per patient (3.6 ± 1.1), followed by Vietnam (2.9 ± 1.5) and lastly, Peru and Bangladesh (2.1 ± 1.9 and 2.1 ± 1.8, respectively) (Additional file 5). The difference between the South African subgroup was significant when compared to the Peru (p = 0.040) and Bangladesh (p = 0.036) subgroups.

**Fig. 3:**
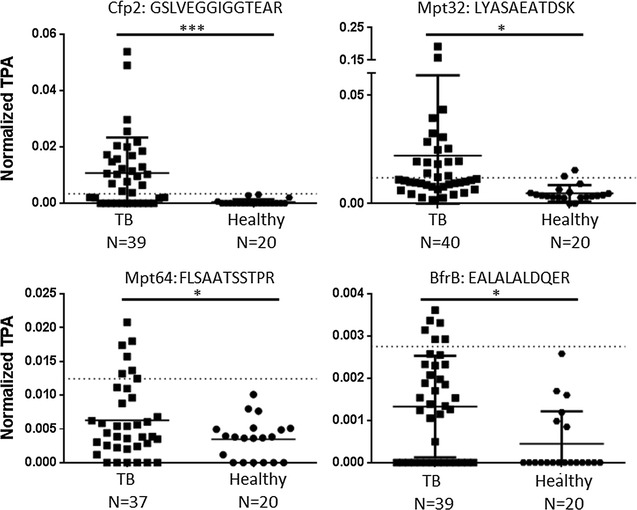
Comparison of normalized total peak areas from the TB patients and healthy individuals. The *solid lines* indicate the mean nTPA with SD. The *dotted line* represents the threshold created by the average normalized value of the healthy plus 3 times the standard deviation; TB patients were designated as positive for a peptide if the TPA was above this threshold. Of the 41 peptides screened, four were statistically more abundant in those with active disease (p values detailed in Additional file 6)

The frequency by which each peptide/protein was identified in our cohort of 40 samples is summarized in Table 4. The top ranking peptide for assay 1 was: GSLVEGGIGGTEAR (from the *Mtb* protein Cfp2); this peptide was identified in 24 out of 40 (60%) samples. Interestingly, this peptide was identified in 90% of all of the South African samples, but in only 50% of the other three locations (Fig. 2). The difference between the normalized TPA in TB patients and healthy individuals is statistically different by *t* test for the GSLVEGGIGGTEAR peptide with a p value < 0.001 (Fig. 3). In addition, the peptides LYASAEATDSK, FLSAATSSTPR, and EALALALDQER from *Mtb* proteins Mpt32, Mpt64, and BrfB, respectively, showed a significant (p < 0.05) difference in signal between those with active disease and the healthy cohort (Fig. 3/Additional file 6). When these 4 significant peptides were stratified by the HIV status of each patient, the normalized TPA values between the HIV positive and HIV negative groups were not significant. Similarly, when stratified by smear status, the differences in signal were not statistically different between groups. One or more of these 4 peptides was represented in 70% of the samples screened. In addition to these four peptides we also identified 16 additional peptides that were detected in at least 1 or up to 7 individual active TB patients and thus also represent candidates for TB disease biomarkers (Additional file 7).

**Table 4:** Percentage of samples positively identified with each *Mtb* peptide (left) and protein (right)

Peptide	Percent (%)	Protein	Percent (%)
GSLVEGGIGGTEAR	60	Cfp2	60
LYASAEATDSK	35	*Mpt32*	38
FLSAATSSTPR	18	Mpt64	18
TAVEQAAAELGDTGR	18	MrsA	18
EALALALDQER	15	BfrB	15
WDATATELNNALQNLAR	15	Esat-6	15
NDPMVQIPR	10	*GroES*	13
RIPLDVAEGDTVIYSK	10	Ag85c	10
IHVEALGGHLTK	10	SahH	10
TTPSIVAFAR	8	Ag85a	8
SVFDDGLAFDGSSIR	8	DnaK	8
NDPLLNVGK	8	GlnA1	8
NYTAPGGGQFTLPGR	5	*GlcB*	8
IALFGNHAPK	5	*AcpM*	5
FALNAANAR	3	PpiA	5
DVLAVVSK	3	Ag85b	3
AELPGVDPDK	3	Cfp10	3
QELDEISTNIR	3	GarA	3
LVFLTGPK	3	HspX	3
IPDEDLAGLR	3		
LEEENPEAAQALR	3		

In bold italics are the proteins represented by more than one peptide, and in each case this results in an additive effect over each peptide considered on its own

Eight peptides in MRM-1 and 9 peptides in MRM-2 showed no discrimination between healthy individuals and those with active tuberculosis (Additional file 8, Panel 1 and 2, respectively). This includes: VQFQGGGPHAVYLLDGLR (Ag85c), FLEGLTLR (Ag85c), AGANLFELENFVAR (BrfB), SLADPNVSFANK (Cfp2), VVADLTPQNQALLNAR (GlcB), TVSLPVGADEDDIK (HspX), SLENYIAQTR (Mpt64), and GVTEETTTGVLR (SahH) from MRM-1 and TVGDVVAYIQK (AcpM), AADMWGPSSDPAWER (Ag85b), ITQDLLDR (DnaK), LAAAWGGSGSEAYQGVQQK (Esat-6), FLLDQAITSAGR (GarA), GGYFPVAPNDQYVDLR (GlnA1), YVLEELR (MrsA), VIQGFMIQGGDPTGTGR (PpiA), and HTIFGEVIDAESQR (PpiA) from MRM-2. As none of these peptides showed a signal above the background level determined by the healthy serum exosomes, these 17 peptides will be excluded as markers of active tuberculosis in future studies. Several of the eliminated peptides correlate with poor LOD values. Since multiple peptides are targeted per protein in our assays, the exclusion of these 17 peptides did not eliminate any of the *Mtb* proteins in our original candidate list. Lastly, three peptides could not be fully evaluated due to the contribution of the matrix effect, as a baseline value in the healthy patient cohort could not be determined (Additional file 9); the suppression of the 3 standard peptides in all 20 healthy exosome samples supports our concern of matrix variation and the use of a pooled matrix for the LOD calculations.

## Discussion

The burden of mycobacterial proteins circulating in human serum during active TB infection is currently unknown, but based on prior proteomic and molecular-based investigations, it is believed to be of low abundance [20]. In this study, we have shown that intracellular mycobacteria contribute to the protein content of exosomes [3, 18] and purification of these microvesicles from the serum can serve as an effective method of depletion of abundant host proteins (e.g. albumin and immunoglobulins) while concentrating the bacterial products of interest. Recent studies have shown that proteins from other mycobacterial species, such as *M. avium* [21], as well as other intracellular pathogens, such as *Helicobacter pylori* [22] can be readily detected in exosomes. In the current study, we sought to confirm and build upon our previous discovery studies in which *Mtb* peptides for 33 unique *Mtb* proteins were detected in human serum or patients with active and/or latent TB [11]. Our previous study utilized 17 unrefined targeted MRM assays, here we optimized the assays to include all selected peptides in two different methods. In this study, we included isotopically labeled standards for the first time, to confirm peptide identification based on retention time and fragmentation pattern while also providing a normalizing tool allowing direct comparison of peak areas of a given peptide from sample to sample. Ideally, we would have included all of the significant peptides determined in our original publication [11], however this was not feasible, due to poor performance of several of the isotope-labeled peptide standards during the development of the MRM assays. Specifically, the Mpt64 protein, dictated the selection of alternative peptides; of the two replacement Mpt64 peptides, one remained statistically significant (Fig. 3), while the second was nondiscriminatory (Additional file 8). Both the addition of the isotope-labeled standards, as well as iRT, retention time standard peptides, provide extra QC tools that allow us to monitor run-to-run variance. Even though RT variability was well within the 2 min monitoring window (Additional file 10), the inclusion of iRTs and labeled standards allowed us to monitor run-to-run variation and to determine the need for re-injection in a few cases were RT shifted across the board due potentially to matrix effects. Monitoring of RT variability also allowed us to see a pattern in which the late eluting peptides seemed to have a more stable RT. Although we did not follow up on the potential reasons for this trend, it may be an important factor to consider during development of future MRMs.

In this study, we also optimized data analysis to increase the stringency of which a given peptide signal is considered positive in any given sample. Matching retention times to those of the labeled standard, as well as the requirement to only count peptides with at least three detected transitions were important parameters in our algorithm. However, for many peptides, the most restrictive filter was the inclusion of the threshold determined by the normalized TPA in the healthy donor samples which represents high noise due to the complexity of the exosome matrix and which could be mistaken for a positive result. Overall, the optimized MRM method including the addition of labeled standards and iRTS and the optimized data analysis algorithm, allowed us to reduce our original candidate biomarker list from 41 to 20 highly significant peptides in the context of active TB disease.

As noted in our method section, the MRM methods presented in this study were optimized with a window of 2 or 4 min for scanning of each peptide. Retention times were optimized using the labeled peptides spiked into a mixture of serum exosomes (our pooled matrix). While for the majority of peptides, the retention time was stable within that window from sample to sample, there are limitations to this type of scheduling, and a few peptides, such as FALNAANAR, AGANLFELENFVAR, VQFQGGGPHAVYLLDGLR and PGLPVEYLQVPSPSMGR, were not routinely detected within our sample window as a result. This is likely because the retention time for these peptides was very dependent on the matrix background and often resulted in either increased or decreased retention time outside of the scanning window. These peptides were also very susceptible to sample-to-sample variation in the total peak area measured for the labeled standard. While scheduling the scanning window for each peptide allowed us to include more peptides in a single method without a reduction in sensitivity, some peptides, such as the ones mentioned above, may benefit from an unscheduled method in which all channels (one for each peptide) are continuously scanned throughout the length of the chromatography. The variation observed here demonstrates the effect that matrix background can have in peptide detection, which was further evidenced by the ion suppression we observed for three labeled peptide standards (TTGDPPFPGQPPPVANDTR, PGLPVEYLQVPSPSMGR, and SVFDDGLAFDGSSIR) in the healthy controls.

Importantly, the variation in retention time and ion suppression described above demonstrates the importance of having isotopically labeled peptides spiked in each sample such that the correct peak is identified (i.e. in cases of retention time drift) and to correctly normalize the area under the peak of the native peptide (i.e. ion suppression dependent on matrix background). The inclusion of isotopically labeled peptides is thus a major improvement from our previous study, strengthening the data set by significantly increasing the confidence for the detection of bona-fide mycobacterial peptides. For future studies, we believe that the methods can be improved even further by optimizing the method without any scheduling and by increasing the number of healthy controls so that more accurate mean TPA and more tolerant cut-offs can be applied as outliers appear to have a significant impact on the threshold.

Blood-based assays are highly desirable for TB diagnostics, as they are independent of an individual’s ability to produce sputum (i.e. children), as well as remove the occupational hazard of transmission during sputum collection. Our study emphasize the benefit of blood based biomarkers by highlighting the prevalence of *Mtb* proteins in serum exosomes in those failed to be identified by the first-line diagnostic screening by sputum microscopy. Downstream translation of our assay to a point-of-care platform is in line with the end goal of creating an assay that detects active infection faster than sputum culture. Of equal importance, the identification of mycobacterial proteins in serum exosomes is not affected by HIV co-infection; serum biomarkers may have an added benefit in detecting disseminated disease often displayed in HIV positive patients.

Inclusion of samples from a variety of geographic locations was another strength of this study, as it provided us with a diverse set of samples from persons with disease caused by different *Mtb* strains [23]. Our original work focused on a clinical cohort from Uganda [11], at this time we did not fully appreciate the potential effect of geography on our results which impacted the selection of protein candidates for this current study. Thus, it is possible that we may have missed proteins or peptides that may perform better in other geographical locations, including those analyzed in the present study. As the mycobacterial contribution to the exosome is reflective of the bacterial secretome, we sought to identify biomarkers common to *Mtb* complex, independent of lineage or strain. However, we found that Cfp2 peptides performed best in the South Africa cohort (90%) and Mpt32 peptides underperformed as a biomarker in Bangladesh (40%, compared to 60–70% in other regions). Cfp2 is a secreted protein for which differences in abundance and secretion among *Mtb* strains had been previously reported by our group [24], supporting the idea that some of the differences we observed among geographical locations may be associated with specific circulating *Mtb* strains. However, other factors such as those related to the host (i.e. matrix effects favoring ionization of specific peptides), as well as the heterogeneity of the infection, may also account for the differences seen here, but additional studies are needed.

## Conclusions

Through improved sample preparation, enhanced MRM assays and optimized data analysis work flow, in this study, we were able to find the proverbial *Mtb* peptide “needle” in a serum “haystack”. Whereas the methodology as it stands is not suitable for the purpose of TB diagnosis in the field setting, it provides confirmation of the presence of mycobacterial protein candidates in serum exosomes. Additional verification and validation studies with larger and more geographically diverse sample sets, as well as translation to a point-of-care assay are future goals.

## Additional files

**Additional file 1.** Table of native and isotope-labeled peptide multiple reaction monitoring parameters including parent and transition m/z, retention time and collision energy.

**Additional file 2.** Limit of Detection/Quantitation (LOD/LOQ) summary for transitions in each peptide in mixed exosome matrix. Values are in fmol/μl.

**Additional file 3.** Table of normalized TPA cut-off values determined by the healthy donor group. False discovery rates (FDR) are calculated as the false positives (healthy controls with nTPA above the cut-off)/total number of healthy controls.

**Additional file 4.** Graph detailing the patient breakdown by smear microscopy and HIV status and the number of peptides identified in each class.

**Additional file 5.** Number of peptides identified per patient by geography.

**Additional file 6.** Statistical analysis summary. Includes results from two-tailed t-test analysis, before and after Benjamin-Hochberg adjustment.

**Additional file 7.** Graphs depicting the sixteen peptides in which several active TB patients displayed TPAs above the healthy threshold; the overall nTPA mean (solids line) between the two groups was not statistically significant.

**Additional file 8.** Summary of seventeen peptides which failed to discriminate between active TB and healthy controls; panel 1 and 2 are the peptides from MRM assay 1 and 2, respectively.

**Additional file 9.** Three peptides for which no cut-off threshold was determined due to indeterminate results in all 20 healthy samples.

**Additional file 10.** A. Peptides retention time (line) and their coefficient of variation (bars) for all samples (darker) and final samples (lighter). Mtb peptides: orange, iRTs: black. B. Boxplot representing retention time deviation from the mean for the 20 Mtb peptides and 9 iRTs. All: includes all samples and replicates including truncated peaks. Final: includes only samples/replicates included in final analysis.

## Abbreviations

MRM: multiple reaction monitoring
MS: mass spectrometry
TB: tuberculosis
Mtb: *Mycobacterium tuberculosis*
FIND: Foundation for Innovative novel Diagnostics
ACN: acetonitrile
FA: formic acid
iRT: indexed retention time
TPA: total peak area
QC: quality control
LOD: limit of detection
LOQ: limit of quantification
FDR: false discovery rate
