# Supplementary figures and images for: Second generation multiple reaction monitoring assays for enhanced detection of ultra-low abundance Mycobacterium tuberculosis peptides in human serum
Source: Clin Proteomics. 2017 Jun 5;14:21. doi: 10.1186/s12014-017-9156-y (PMC5460347; doi:10.1186/s12014-017-9156-y)

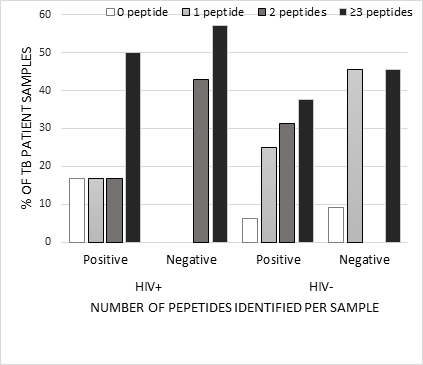

Supplement: Supplementary file 4 — Additional file 4. Graph detailing the patient breakdown by smear microscopy and HIV status and the number of peptides identified in each class. [file 12014_2017_9156_MOESM4_ESM.tif]

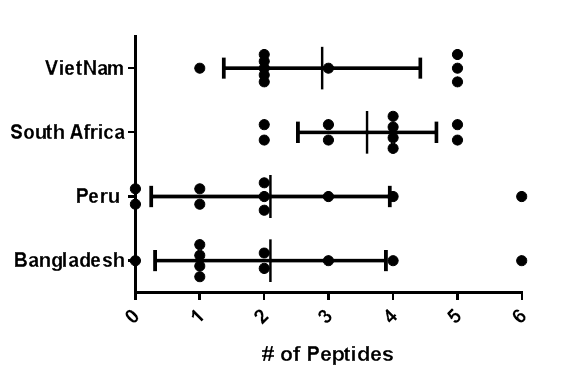

Supplement: Supplementary file 5 — Additional file 5. Number of peptides identified per patient by geography. [file 12014_2017_9156_MOESM5_ESM.tif]

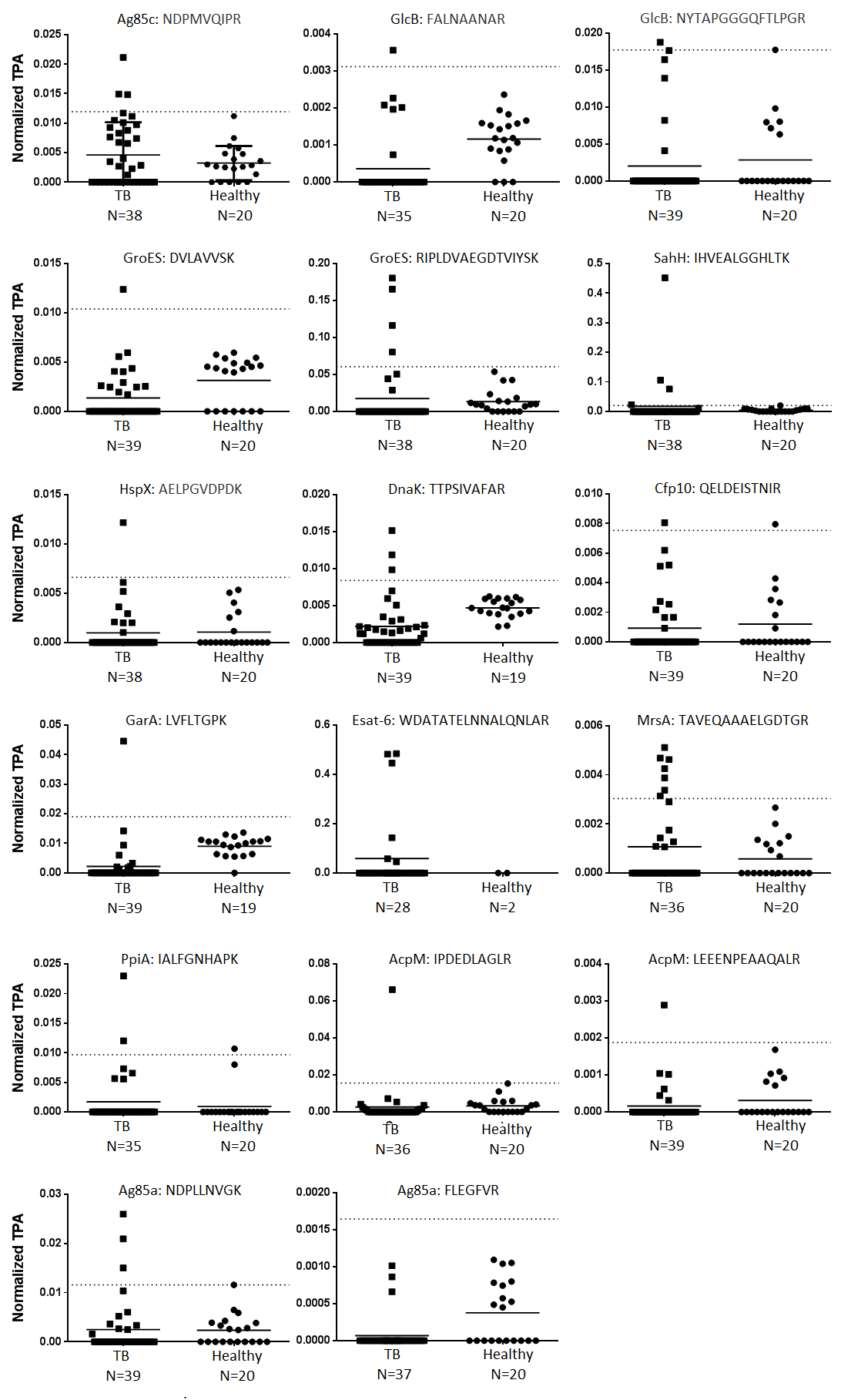

Supplement: Supplementary file 7 — Additional file 7. Graphs depicting the sixteen peptides in which several active TB patients displayed TPAs above the healthy threshold; the overall nTPA mean (solids line) between the two groups was not statistically significant. [file 12014_2017_9156_MOESM7_ESM.tif]

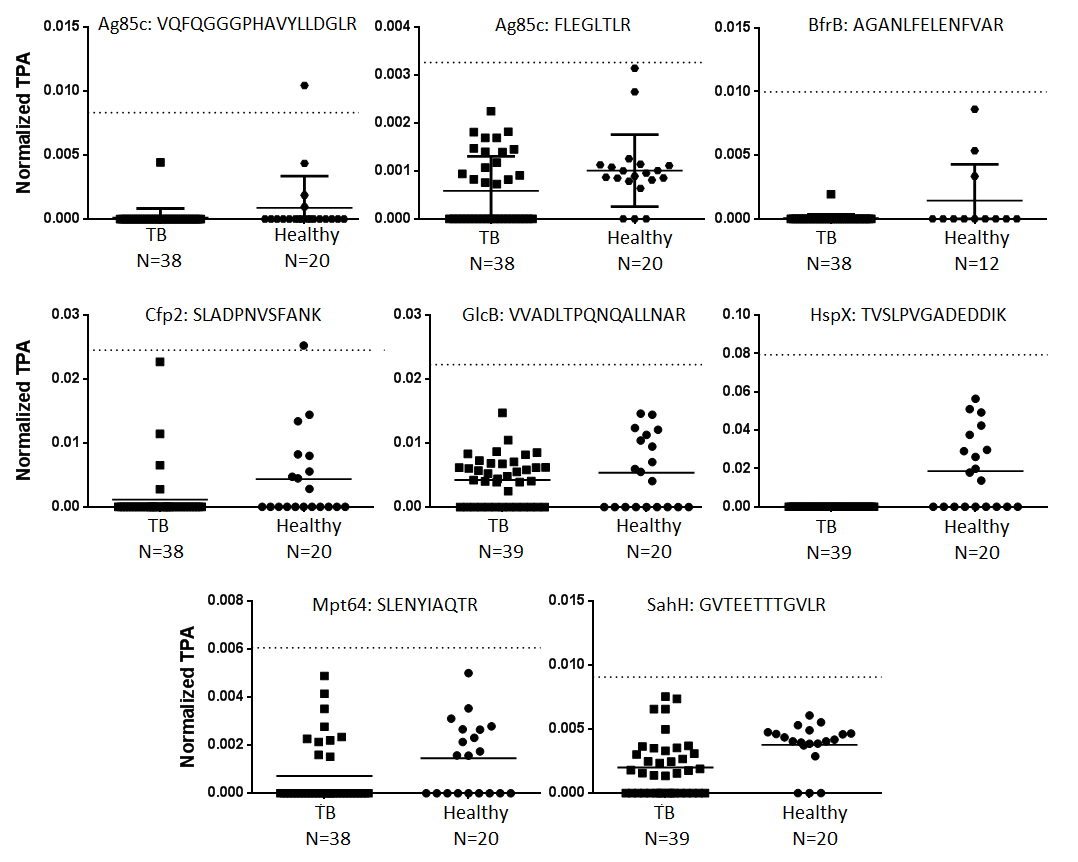


Additional file 8 panel1


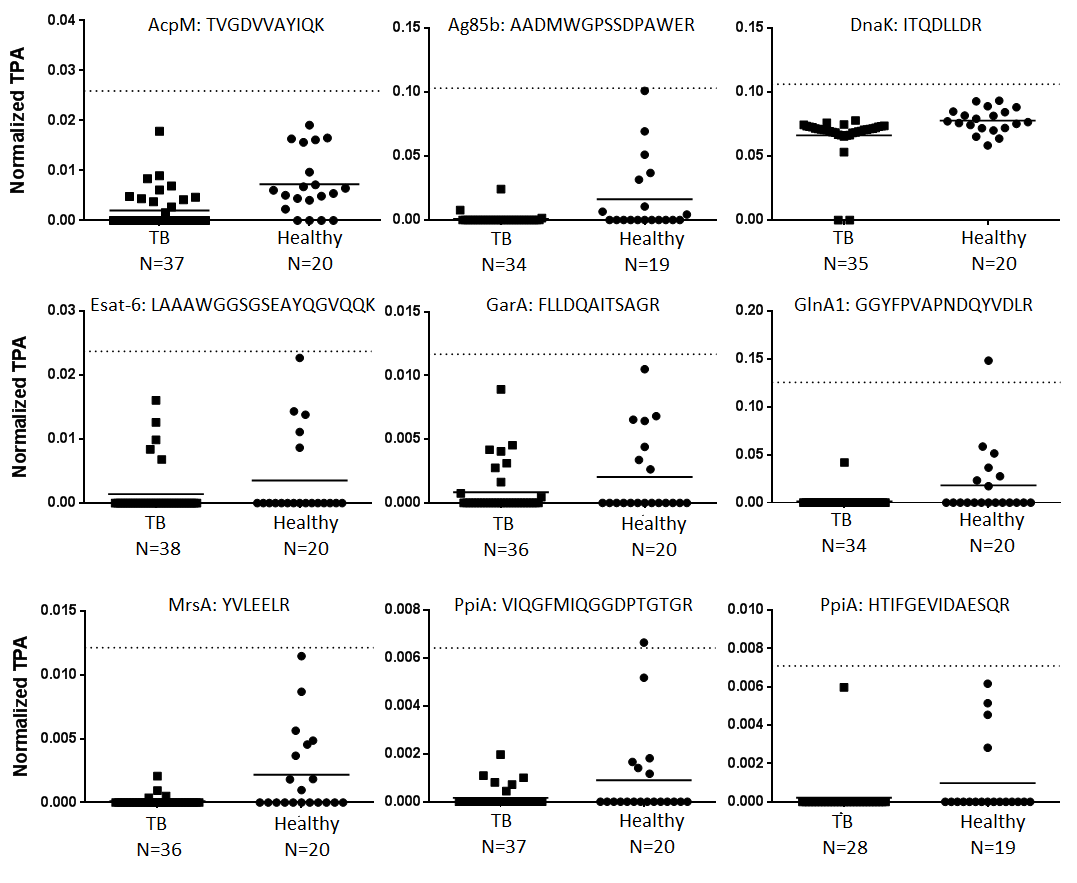


Additional file 8 panel2

Supplement: Supplementary file 8 — Additional file 8. Summary of seventeen peptides which failed to discriminate between active TB and healthy controls; panel 1 and 2 are the peptides from MRM assay 1 and 2, respectively. [file 12014_2017_9156_MOESM8_ESM.doc]

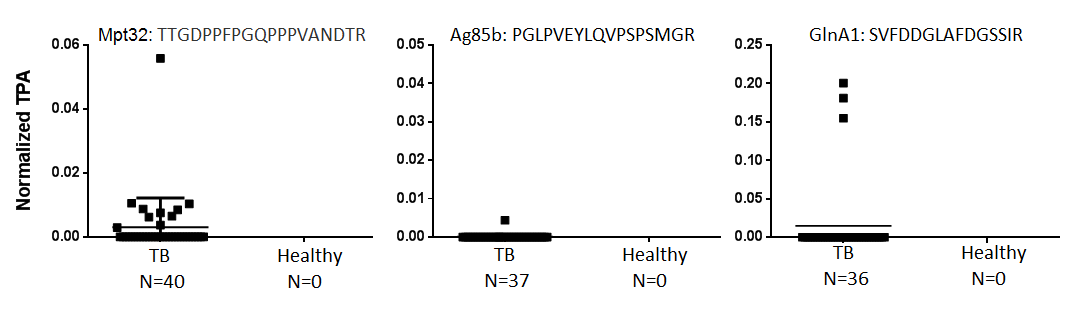

Supplement: Supplementary file 9 — Additional file 9. Three peptides for which no cut-off threshold was determined due to indeterminate results in all 20 healthy samples. [file 12014_2017_9156_MOESM9_ESM.tif]

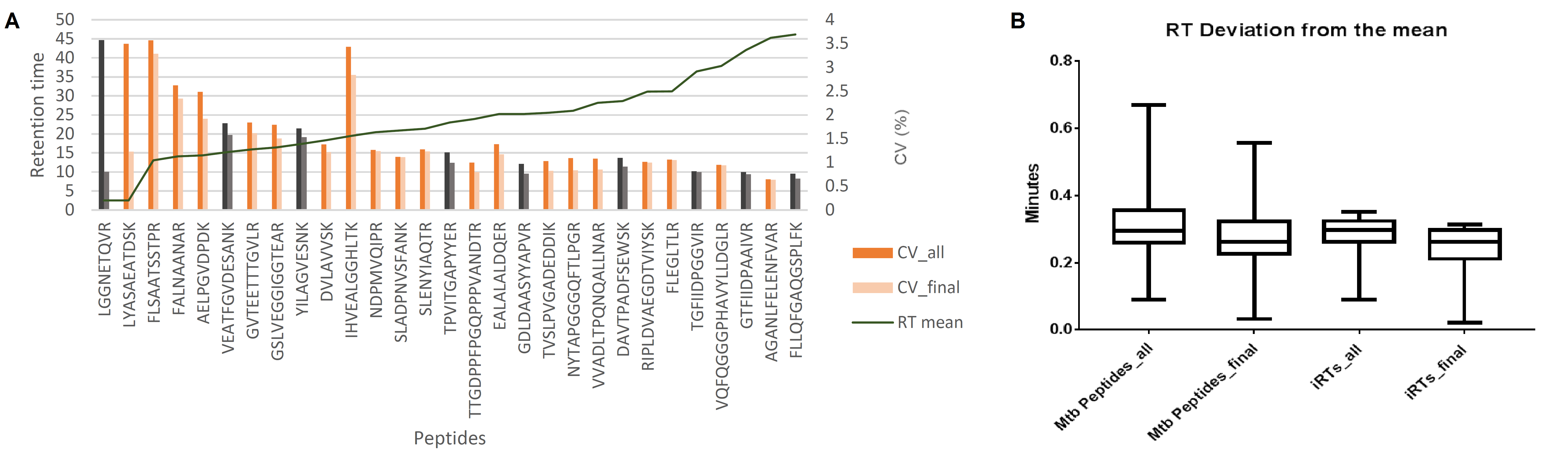

Supplement: Supplementary file 10 — Additional file 10. A. Peptides retention time (line) and their coefficient of variation (bars) for all samples (darker) and final samples (lighter). Mtb peptides: orange, iRTs: black. B. Boxplot representing retention time deviation from the mean for the 20 Mtb peptides and 9 iRTs. All: includes all samples and replicates including truncated peaks. Final: includes only samples/replicates included in final analysis. [file 12014_2017_9156_MOESM10_ESM.tif]
